# The effect of mouthwashes containing biguanides on the progression of erosion in dentin

**DOI:** 10.1186/1472-6831-14-131

**Published:** 2014-10-30

**Authors:** Senda Charone, Cristiane de Almeida Baldini Cardoso, Melissa Thiemi Kato, Paula Ducati, Rejane Fukushima, Gabriela Gennaro, Ana Carolina Magalhães, Marília Afonso Rabelo Buzalaf

**Affiliations:** Department of Biological Sciences, Bauru School of Dentistry, University of São Paulo, Al. Octávio Pinheiro Brisolla, 9-75, Bauru, SP 17012-901 Brazil; Department of Dentistry, University of Sagrado Coração, Bauru, Brazil; Department of Consumer and Market Knowledge, Hypermarcas, Kragujevac, Brazil

**Keywords:** Biguanide, Demineralization, Dentin, Erosion

## Abstract

**Background:**

Dental erosion is caused by frequent exposure to acids without the involvement of microorganism. This study analyzed the effect of biguanides (polyhexamethylene biguanide – PHMB and chlorhexidine – CHX) on dentin erosion due to their possible influence on the enzymatic degradation of the demineralized organic matrix.

**Method:**

Sixty bovine dentin specimens were prepared. On both sides of their surface, nail varnish was applied to maintain the reference surfaces for the determination of dentin loss. Samples were cyclically de- and remineralized for 6 days. Demineralization was performed with a 0.87 M citric acid solution (6×5 min daily). Thereafter, samples were treated with distilled water (negative control), 0.12% CHX (positive control), 0.07% PHMB, Sanifill Perio Premium™ (0.07% PHMB plus 0.05% NaF), or F solution (0.05% NaF) for 1 min and then subjected to enzymatic challenge for 10 min using a bacterial collagenase (*Clostridium hystoliticum*, 100 μg/ml). Dentin loss was assessed using profilometry (μm) daily. Data were analyzed using 2-way repeated measures-ANOVA and Bonferroni’s test (p < 0.05).

**Results:**

Dentin loss progressed significantly for all groups during the 6 days. After the 3^rd^ day, Sanifill Premium™, CHX, and PHMB significantly reduced dentin erosion compared to control. On the 6^th^ day, the lowest mean (±SD) dentin loss was observed for Sanifill Perio Premium™ (94.4 ± 3.9 μm). PHMB and CHX led to intermediate dentin loss (129.9 ± 41.2 and 135.3 ± 33.5 μm, respectively) that was significantly lower than those found for negative control (168.2 ± 6.2 μm). F (157.4 ± 6.1 μm) did not significantly differ from negative control.

**Conclusions:**

Sanifill Perio Premium™ mouthwash has a good potential to reduce dentin loss, which might be associated with the presence of PHMB.

## Background

Dental erosion is caused by frequent exposure to exogenous or endogenous acids without the involvement of microorganism [1]. Nowadays this clinical condition is in evidence because people are more often exposed to acids, and researchers give more attention to the early diagnosis. Consequently, increment in prevalence has been reported [2, 3].

When enamel is lost or the root is exposed because of gingival recession, dentin can be accomplished by this condition. Dentin erosive tissue loss is not a simple surface process. The minerals from the peritubular dentin are initially released, and the tubules are widened [4]. Subsequently, a superficial layer of demineralized organic matrix (DOM) is detectable. It is conceivable that DOM might protect the remaining demineralized dentin against mechanical forces such as toothbrush abrasion [5, 6]. In addition, DOM (mainly collagen fibrils) is able to hamper ionic diffusion into and out of the demineralized area, modulating the progression of mineral loss [7, 8]. However, DOM may be affected by enzymatic and chemical degradation, which might enhance the progression of dentin erosion [9, 10].

DOM may be affected by host-derived enzymes, such as MMPs, which mediate the breakdown of practically all extracellular matrix molecules, including native and denatured collagen [11, 12]. MMP-8 has been found to be the most common collagenase of human dentin [13], but MMP-2 and MMP-9 have also been found in this tissue [14, 15]. It has been shown that the activity of MMP-2 and MMP-9 is crucial for dentin collagen breakdown in carious lesions [16]. However, the degradation of DOM in vivo does not depend only on the action of MMPs. Other proteases have been recently suggested to be implicated in this process such as cysteine cathepsins [17]. Cysteine cathepsins can activate MMPs [18], and this mechanism has been suggested to be involved in human dentinal caries lesions [19]. Cysteine cathepsins are derived from the dental pulp via dentinal fluid and can cleave C-terminal telopeptide of type I collagen [20], possibly exposing the collagenase cleavage site, after the exposure to the acids. Besides, it has been shown that cathepsin B directly cleaves and inactivates MMP-specific tissue inhibitors TIMP-1 and TIMP-2, changing the balance between MMPs and their inhibitors. Acidic pH activates cysteine cathepsins, which in turn either activate MMPs or inactivate TIMP, or both, resulting in degradation of DOM [21].

Collagenase inhibitors are being investigated as potential therapeutic agents in the treatment and prevention of several oral conditions, including dentin caries and erosion [22–24]. Chlorhexidine (biguanide, CHX), an MMP inhibitor [25] and cysteine cathepsin inhibitor [26], has been suggested as a useful tool in the prevention of collagen fibrils degradation by collagenases, thus increasing the longevity of adhesive restorations [27–29] as well as reducing the erosion progression [30, 31].

Polyhexamethylene biguanide hydrochloride (PHMB) is a polymeric biguanide with broad antimicrobial spectrum against both Gram-positive and Gram-negative bacteria [32, 33] and has been used for several years as an antiseptic agent in medicine [34]. PHMB is a structural analogue of chlorhexidine, with the advantage of not having side effects such as tooth staining. We hypothesized that PHMB could reduce dentin erosion, as chlorhexidine, by inhibiting collagenases.

Taking this into consideration, this study analyzed the effect of biguanides (PHMB and CHX) on dentin erosion. The null hypothesis tested was that 0.07% PHMB would not reduce erosion progression compared to 0.12% CHX.

## Methods

### Sample preparation

Sixty dentin samples (4 × 4 × 3 mm) were cut from extracted sound bovine incisor crowns using an ISOMET Low Speed Saw cutting machine (Buehler Ltda., Lake Bluff, IL, USA) and two diamond disks (Extec Corp., Enfield, CT, USA), which were separated by a 4-mm thickness spacer. The enamel surface was removed using a diamond bur (KG Sorensen 4054, Cotia, SP, Brazil), and then the dentin surface was ground flat with water-cooled carborundum discs (600 and 1200 grades of Al_2_O_3_ papers; Buehler, Lake Bluff, IL, USA) and polished with felt paper wet with diamond suspension (1 μm; Buehler, Lake Buff, IL, USA). The protocol was approved by the Institutional Review Board of Bauru Dental School, University of São Paulo.

Dentin samples were randomly distributed into five groups (n = 12): distilled water (negative control), 0.12% CHX (positive control), 0.07% PHMB, Sanifill Perio Premium™ (0.07% PHMB plus 0.05% NaF), or F solution (0.05% NaF).

Two layers of nail varnish were applied on two-thirds of the outer dentin surface, in order to maintain reference surfaces for dentin-loss determination.

### Treatment and erosive challenges

All samples were subjected to de- and remineralization cycles for 6 days. Six demineralization challenges were performed per day for 5 min each, using 0.87 M citric acid solution (pH 2.5, 30 ml/sample). After each demineralization, samples were treated with one of the solutions for 1 min (30 ml/sample). Thereafter, they were rinsed in deionized water and then subjected to enzymatic challenge for 10 min using a bacterial collagenase (*Clostridium hystoliticum*, 100 μg/ml, Sigma-Aldrich, EUA) in a buffer containing 1 ml of 50 mM TES buffer (TESCA), pH 7.4 [35, 36]. Between the cycles, the samples were immersed in artificial saliva (1.5 mmol l^-1^ CaCl_2_, KH_2_PO_4_ 2.4 mmol l^-1^, Na_2_HPO_4_, 17 mmol l^-1^ KCl, 2 mmol l^-1^ NaSCN, 9.9 mmol l^-1^ NaCl_,_ 3 mmol l^-1^ NH_4_Cl_,_ 3 mmol l^-1^ urea, and 0.2 mmol l^-1^ glucose, pH 6.8, 30 ml/sample).

### Dentin loss measurement

Dentin loss was assessed using a contact profilometer (MarSurf GD 25, Mahr, Göttingen, Germany) daily. For this purpose, the dentin samples were kept moist in 0.9% NaCl and 0.02% sodium azide solution at 4°C to avoid shrinkage of the DOM during the measurement. Firstly, the nail varnish over the reference surfaces was carefully removed using a scalpel blade and acetone solution (1:1 water). The diamond stylus moved from one reference area to the exposed one and then over the other reference area (length: 2.5 mm). The dentin profile was determined by comparing the reference surfaces to the eroded area using an appropriate software (MarSurf XT 20, Mahr). The differences in height between the reference and eroded areas were quantified in microns. Five profile measurements were performed at a distance of 250 μm from each other and the average value obtained.

### Statistical analysis

The software GraphPad Prism (GraphPad Software, San Diego, USA) was used. Data were checked for normality and homogeneity using Kolmogorov-Smirnov and Bartlett’s tests, respectively. Since these criteria were satisfied, data were analyzed using 2-way repeated measures ANOVA followed by Bonferroni’s post hoc test considering treatments and periods as factors (p < 0.05).

## Results

Dentin erosive loss progressed significantly for all groups during the 6 days. The highest and the lowest progression were found for dentin samples from the control and Sanifill Perio Premium™ groups, respectively. On the 1^st^ day, no differences were found among the groups, while on the 2^nd^ day, CHX and Sanifill Perio Premium™ were able to significantly reduced dentin loss compared to control. On the 3^rd^ day, only Sanifill Perio Premium™ was able to significantly reduce dentin erosion.

From the 3^th^ to the 6^th^ day, the lowest mean (±SD) dentin loss was still observed for Sanifill Perio Premium™, which significantly differed from all the other groups (except from CHX on day 4). PHMB and CHX led to intermediate dentin loss values that were significantly lower than those found for control. F alone did not significantly differ from control along the experimental days (Table 1).

**Table 1:** **Dentin loss (μm, mean ± SD) for each treatment group after the experimental days**

Group	Day 1	Day 2	Day 3	Day 4	Day 5	Day 6
**Distilled water**	52.6 ± 4.7	82.2 ± 3.4	109.2 ± 2.5	135.4 ± 6.0	157.8 ± 55.2	168.2 ± 6.2
	^Aa^	^Bc^	^Cbc^	^Dd^	^Ed^	^Ec^
**CHX**	43.6 ± 5.1	54.1 ± 2.6	92.1 ± 2.8	96.5 ± 2.8	111.7 ± 3.9	135.3 ± 33.5
	^Aa^	^Aab^	^Bb^	^BCab^	^Cb^	^Db^
**PHMB**	41.7 ± 2.7	68.7 ± 1.9	96.3 ± 2.2	109.1 ± 3.9	121.1 ± 4.7	129.9 ± 41.2
	^Aa^	^Bbc^	^Cb^	^Cbc^	^CDbc^	^Db^
Sanifill Perio Premium™	46.0 ± 6.3	46.2 ± 3.0	53.8 ± 4.1	87.6 ± 4.3	89.1 ± 2.3	94.4 ± 3.9
	^Aa^	^Aa^	^Aa^	^Ba^	^Ba^	^Ba^
**F solution**	50.4 ± 7.1	79.3 ± 2.5	119.5 ± 3.3	128.7 ± 3.3	146.0 ± 39.4	157.4 ± 6.1
	^Aa^	^Bc^	^Cc^	^Ccd^	^Dcd^	^Dc^

Treatments whose mean ± SD are followed by distinct lower case letters at the same column differ significantly (p < 0.0001). Days whose mean ± SD are followed by distinct upper case letters at the same line differ significantly (p < 0.0001). There was interaction between the factors (p < 0.0001).

## Discussion

Antimicrobial mouthwashes containing biguanide have been used for a long time [37, 38] to help the treatment of gingivitis and periodontitis and to favor the reduction of dental caries [38]. However, other benefits have been proposed with the frequent use of biguanide solutions (CHX), such as the prevention of the degradation of DOM and consequently of the progression of dental erosion [30].

Considerable evidence supports the importance of MMPs and cysteine cathepsins as key host cell-derived mediators of the pathological tissue destruction observed during periodontitis, cancer, and caries [21, 24, 25, 39–41]. It is well known that MMPs activation occurs at a low pH and that their degrading activity is enhanced by the subsequent pH neutralization, which occurs in caries and erosion processes [16, 21, 42]. In the present study, demineralization was performed using a citric acid solution (an extrinsic erosive acid). The DOM was treated and then exposed to collagenase, simulating the host-derived activated MMPs. The mechanism of action of MMPs has been suggested to be involved with the aid of cysteine cathepsins. Acidic pH activates cysteine cathepsins, which in turn either proteolytically activate MMPs or inactivate TIMP, or both, resulting in degradation of DOM [19, 21]. Thus, the impact of the inhibitory effect of CHX and PHMB on MMPs and cysteine cathepsins and, consequently, on the progression of dentin erosion needs to be better investigated.

Considering the anti-MMPs potential of CHX [25, 30] and that PHMB is a structural analogue of CHX, this study contributes to the field of research by showing that PHMB can be as effective as CHX on the dentin erosion inhibition. Therefore, the null hypothesis can be rejected.

CHX is a cationic biguanide with broad-spectrum antimicrobial action, whose effectiveness in decreasing the formation of dental biofilm (plaque) and gingivitis has been demonstrated in clinical studies [43, 44]. An important characteristic of chlorhexidine is its substantivity or persistence of action, which consists of the ability of this product to bind to oral tissues and remain active for long periods after application. The antimicrobial properties of mouthwashes containing CHX and other antimicrobial agents have been assessed *in vivo* and *in vitro,* with excellent results for CHX-based solutions [44].

CHX was administered in a commercial dose, exceeding those reported for MMP-2, MMP-8, and MMP-9 inhibition [25]. It has been demonstrated that CHX completely inhibits MMP-2 and MMP-9 at a concentration of 0.03% and MMP-8 at a concentration of 0.02–0.01% [28]. There is no data reporting that the inhibitory dose of CHX or PHMB is able to act on the cathepsines or that the PHMB inhibitory dose is able to act on different MMPs, which should be tested in the future.

An interesting finding is that the commercial product Sanifill Perio Premium™, containing PHMB and fluoride, had the greatest dentin erosion inhibiting-effect. This might be due to the combination of both agents, but especially to the presence of PHMB, as confirmed by the results of the PHMB and F groups.

The efficacy of F to reduce dentin erosive demineralization was shown previously [45]. However, this efficacy seems to be highly dependent on the presence of DOM [9, 46, 47]. The present study confirms previous findings, since F solution alone was ineffective to reduce dentin erosion progress, using this experimental model with collagenolytic degradation, where DOM was degraded.

It has been assumed that DOM exhibits a buffering capacity which might prevent the acid attack of deep dentin areas and reduce further demineralization in the presence of high amounts of fluoride [9]. Besides the effect of F inhibiting demineralization, Kato et al. [48] recently showed the ability of NaF to completely inhibit the activity of MMPs. The authors showed that the inhibition of MMPs-2 and -9 by NaF is reversible at low, but irreversible at high F concentrations (5,000 ppm). The possible mechanism by which NaF inhibits the MMPs is not known so far. Considering that MMPs are Zn^2+^ and Ca^2+^ dependent enzymes, and F is highly electronegative, it seems logical that excess F could make these cations unavailable to participate in the catalytic process. In the present study, the F concentration was much lower than those tested by Kato et al. [48], which could help to explain the absence of the erosion inhibiting-effect.

Despite a lack of studies evaluating the role of MMPs in dental erosion, it might be speculated that similar processes to those in caries might affect the degradation of the dentinal organic matrix also under erosive conditions. As the maintenance of the organic matrix is desirable to decrease the progression of erosive dentin lesions [9], it seems worthy to analyze whether the application of potential MMP-inhibitors indirectly affects the progression of erosion by reducing the organic breakdown. Previous studies found that the application of potential MMP-inhibitors reduced the dentin collagen solubilisation [49] and diminished the progression of caries lesions in rats [24]. One potent MMP-inhibitor affecting MMP-2, -8, and -9 is chlorhexidine [25], which was shown to reduce the degradation of the dentin hybrid layer and the solubilisation of dentinal collagen [49].

The inhibitory effect of chlorhexidine on MMPs (zinc-activated, calcium-dependent endopeptidase) is attributed to a chelating mechanism, since the inhibition of MMP-2 and MMP-9 could be prevented by the addition of calcium chloride-binding chlorhexidine. It was also discussed that chlorhexidine might affect essential sulfhydryl groups and/or cysteine present in the active site of MMPs [25]. At salivary concentrations above 0.2%, the inhibitory action of chlorhexidine might be also related to a protein denaturation [50], which might be not the case in the present study.

PHMB present in Sanifill Perio Premium™ might have acted against DOM degradation by similar mechanism of action as CHX. However, additional studies more closely resembling the clinical situation and using different response variables should be conducted to clarify this point.

## Conclusion

In conclusion, both biguanides were able to reduce dentin erosion. Sanifill Perio Premium™ mouthwash had the best potential to reduce dentin erosion, which might be due to the combination of PHMB and F. Further studies must be performed to confirm the mechanism of action of the PHMB-based products.
